# Supplementary figures and images for: Crystal structure of the kinase domain of a receptor tyrosine kinase from a choanoflagellate, Monosiga brevicollis
Source: PLoS One. 2023 Jun 13;18(6):e0276413. doi: 10.1371/journal.pone.0276413 (PMC10263333; doi:10.1371/journal.pone.0276413)

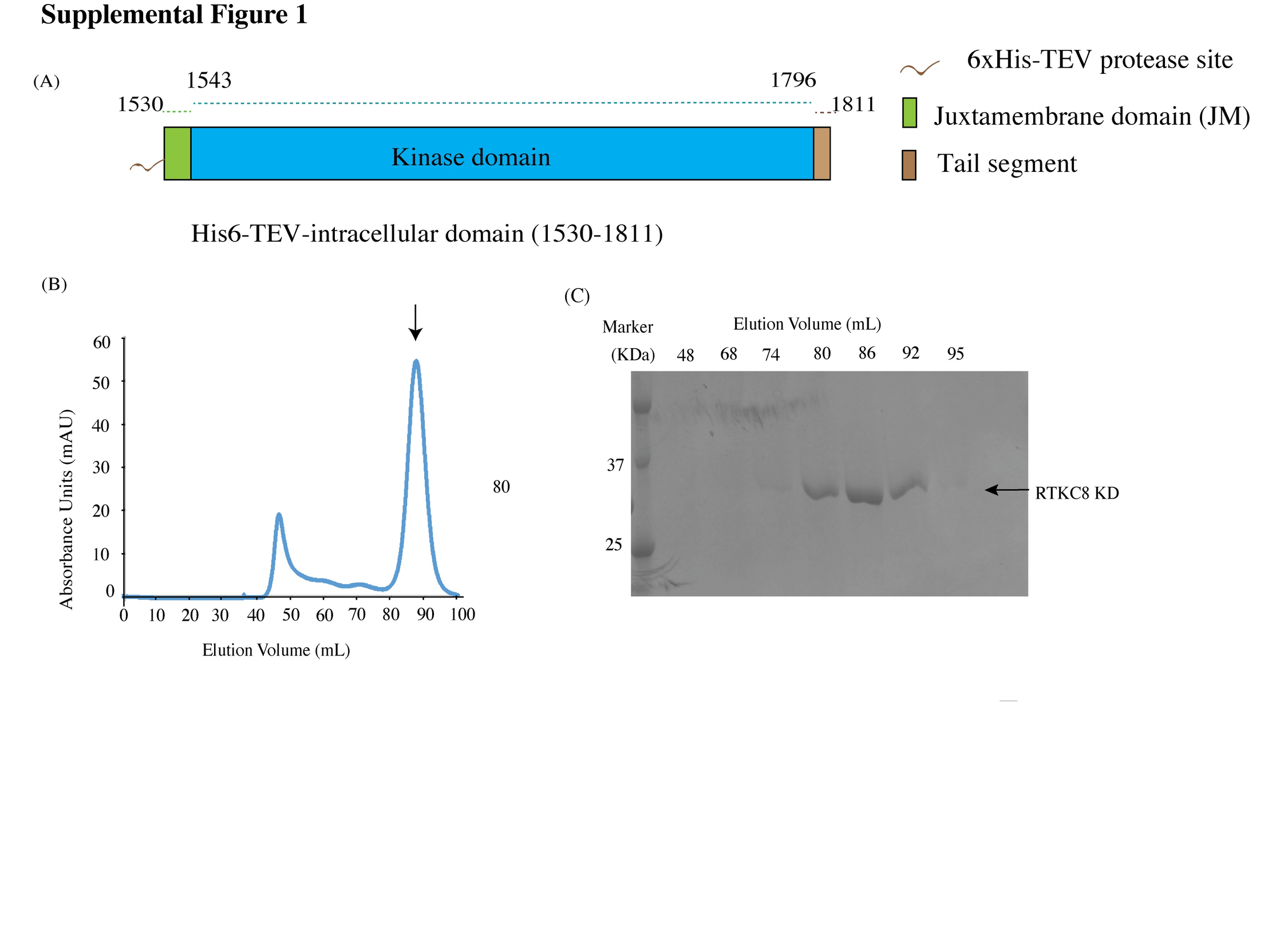

Supplement: S1 Fig — (A). The expressed construct consists of an N-terminal hexa-His tag followed by a TEV protease site and the intracellular kinase domain flanked by residues from the juxtamembrane and tail (RTKC8 residues 1530–1811). (B) Chromatogram showing the protein eluted from gel filtration (Superdex 200 16/600 column) as a single symmetric peak at the expected elution volume for a monomer. (C) SDS-PAGE gel of fractions from gel filtration confirmed the protein purity. (TIF) [file pone.0276413.s001.tif]

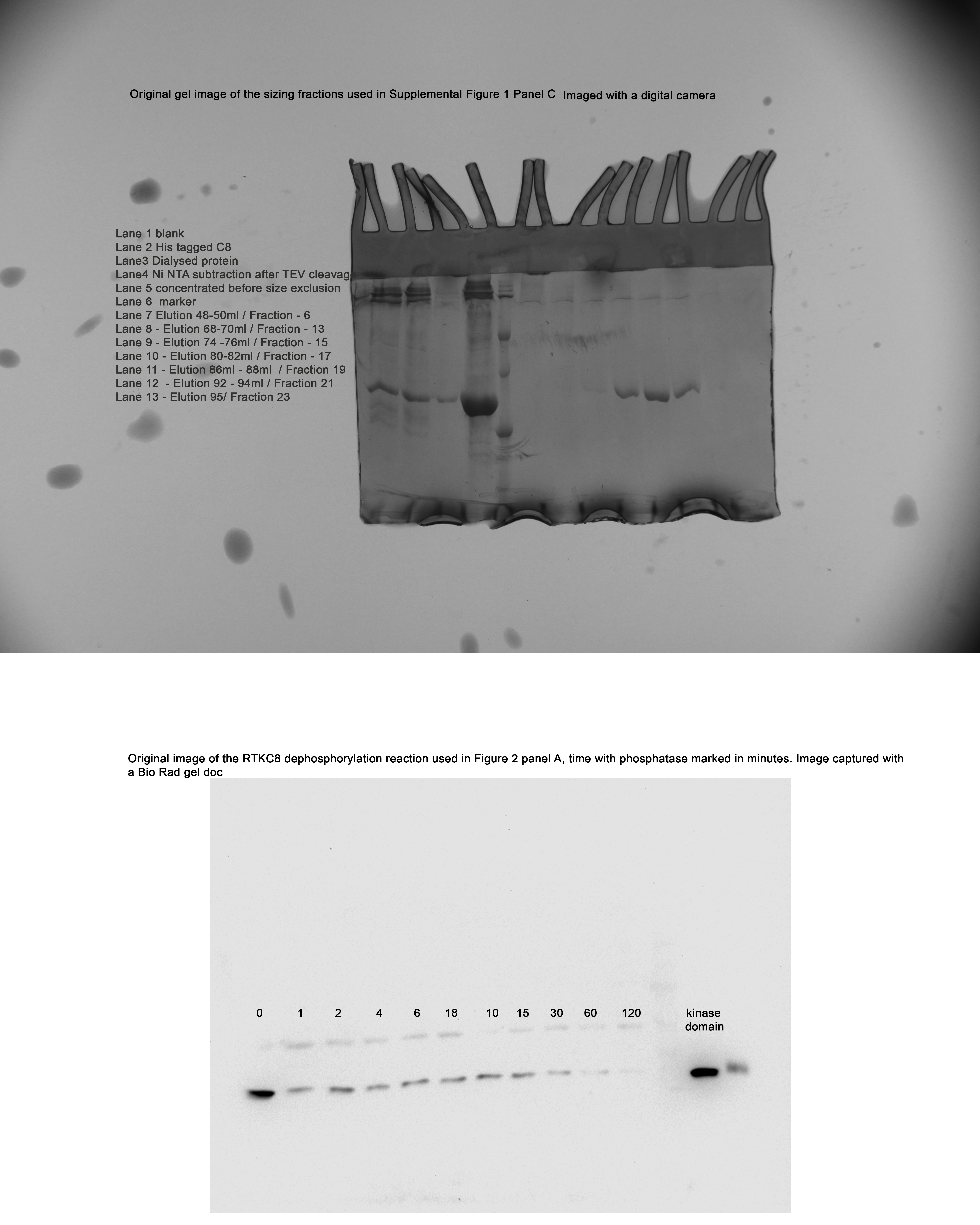

Supplement: S1 Raw images — (TIF) [file pone.0276413.s002.tif]
